# Descriptive and Functional Genomics in Neonatal Respiratory Distress Syndrome: From Lung Development to Targeted Therapies

**DOI:** 10.3390/ijms25010649

**Published:** 2024-01-04

**Authors:** Mădălina Anciuc-Crauciuc, Manuela Camelia Cucerea, Florin Tripon, George-Andrei Crauciuc, Claudia Violeta Bănescu

**Affiliations:** 1 Genetics Department, George Emil Palade University of Medicine, Pharmacy, Science, and Technology, 540142 Târgu Mureș, Romania; madalina.anciuc@umfst.ro (M.A.-C.); claudia.banescu@umfst.ro (C.V.B.); 2 Neonatology Department, George Emil Palade University of Medicine, Pharmacy, Science, and Technology, 540142 Târgu Mureș, Romania; manuela.cucerea@umfst.ro; 3 Genetics Laboratory, Center for Advanced Medical and Pharmaceutical Research, George Emil Palade University of Medicine, Pharmacy, Science, and Technology, 540139 Târgu Mureș, Romania; andrei.crauciuc@umfst.ro

**Keywords:** neonatal respiratory distress syndrome (RDS), pulmonary surfactant, lung development, surfactant synthesis genes, therapeutic targets

## Abstract

In this up-to-date study, we first aimed to highlight the genetic and non-genetic factors associated with respiratory distress syndrome (RDS) while also focusing on the genomic aspect of this condition. Secondly, we discuss the treatment options and the progressing therapies based on RNAs or gene therapy. To fulfill this, our study commences with lung organogenesis, a highly orchestrated procedure guided by an intricate network of conserved signaling pathways that ultimately oversee the processes of patterning, growth, and differentiation. Then, our review focuses on the molecular mechanisms contributing to both normal and abnormal lung growth and development and underscores the connections between genetic and non-genetic factors linked to neonatal RDS, with a particular emphasis on the genomic aspects of this condition and their implications for treatment choices and the advancing therapeutic approaches centered around RNAs or gene therapy.

## 1. Introduction

Based on the information provided by the World Health Organization (WHO), annually across the globe, an estimated 15 million infants are born prematurely [1]. Neonatal respiratory distress syndrome (RDS) remains the leading cause of respiratory failure in premature neonates [2]. Despite advancements, respiratory failure continues to be the primary cause of mortality in early infancy [3].

Moreover, even with the development of modern medical interventions, it is essential to acknowledge that not all infants born at the same gestational age exhibit identical responses to these treatments.

Understanding the fundamental mechanisms, clinical presentations, diagnostic techniques, preventative measures, and treatment approaches for this condition is of utmost significance in lowering morbidity and mortality. In this review, we aimed to highlight the genetic and non-genetic factors associated with RDS while also focusing on the genomic aspect of this condition. Additionally, the following sections will address the treatment options and the progressing therapies based on RNAs or gene therapy.

## 2. Methods

To conduct this review, we performed an extensive and systematic search for relevant references indexed in the Pubmed database up to October 2023, using the following keywords: “neonatal respiratory distress syndrome”, “lung development” “pulmonary surfactant”, “surfactant synthesis genes”, “surfactant synthesis epigenetics”, and “therapeutic targets”. Additional searches, mainly for variant classification but also for related articles, were performed on the Varsome, ClinVar, and Ensembl genome browsers.

Furthermore, we examined the bibliographies of the incorporated articles to uncover additional pertinent publications that align with the objectives of this paper. Following this, we scrutinized the complete texts of each article included in this review. It is worth noting that we opted to include only articles written in English, which could be considered a potential limitation of our study.

We assessed 161 publications for relevance and incorporated them into our current study. Among these, 89 presented original content, 33 were review articles, 4 were editorials, and 25 were case reports. Among the original articles, the majority featured both in vitro and in vivo experiments. Specifically, 2 of the in vivo studies employed inorganic nanomaterials.

Background information on the specific lung development stages or embryology of the respiratory system has been incorporated by referencing older publications, contributing to a contextual understanding of recent discoveries.

## 3. Human Lung Development

Neonatal Respiratory Distress Syndrome (RDS) develops from a shortage of surfactant, stemming from either inadequate surfactant production or, primarily, its inactivation in underdeveloped lungs, often associated with fetal immaturity. Prematurity impacts both of these aspects, consequently playing a direct role in developing RDS [4]. Therefore, it is crucial to analyze the progress of fetal lung development and surfactant production in order to comprehend the fundamental origins of RDS.

The formation of the lower respiratory tract commences around day 22 of fetal development. This intricate process unfolds through five distinct stages, discussed below. During the development of the lungs, precursor cells that line the embryonic lung tubes undergo differentiation into various cell types, exhibiting variation along the cephalo–caudal axis of the lung. These distinct types of epithelial cells play roles in processes such as mucociliary clearance, the transport of fluids and electrolytes, the production of innate host defense molecules, and the synthesis of pulmonary surfactant, which is crucial for postnatal gas exchange. Irregularities in the proliferation and differentiation of these epithelial cells are linked to both acute and chronic lung conditions, including neonatal respiratory distress syndrome, bronchopulmonary dysplasia (BPD), chronic obstructive pulmonary disease, congenital cystic adenomatoid malformation, cystic fibrosis, tracheal–esophageal fistula, tracheal/lung agenesis, and lung tumor development [5]. At the same time, fetal respiratory movements (FBMs) are suggested to contribute to pulmonary development by regulating both lung growth and cell differentiation, as the morphological differentiation of both type I and type II cells seems unattainable in the absence of respiratory muscles. Simultaneously, the brainstem’s mesencephalic Kolliker–Fuse nucleus rhythmically disinhibits the intermediolateral nucleus of the spinal cord, influencing FBMs and potentially explaining surfactant release. The interplay between mechanical stimuli, brainstem activity, and surfactant dynamics underscores their collective contribution to lung development [4].

A comprehension of the genes and mechanisms governing the maturation of the respiratory epithelium has established a foundation for diagnosing and treating disorders impacting perinatal lung function.

### 3.1. Lung Development Stages

#### 3.1.1. Embryonic Stage: 4–7 Weeks—Organogenesis

The initiation of lung development occurs during the third week of gestation, when a small outgrowth emerges from the ventral wall of the foregut. This outgrowth, known as the primitive respiratory diverticulum or lung bud, extends in a caudal direction within the adjacent mesoderm, growing anteriorly and parallel to the primitive esophagus [6]. Over a few days, the gap between the diverticulum and the foregut closes, leaving the only connection at the location where the hypopharynx and larynx will eventually form [7]. On gestational day 28, the respiratory diverticulum undergoes a first division, giving rise to the primary bronchial buds or main stems, which branch into the right and left sides [8]. Subsequently, a second phase of branching takes place approximately during the fifth week, leading to the emergence of secondary bronchial buds. Lastly, the third branching event occurs around the sixth week after conception, resulting in the development of tertiary bronchi [9].

**Figure 1:**
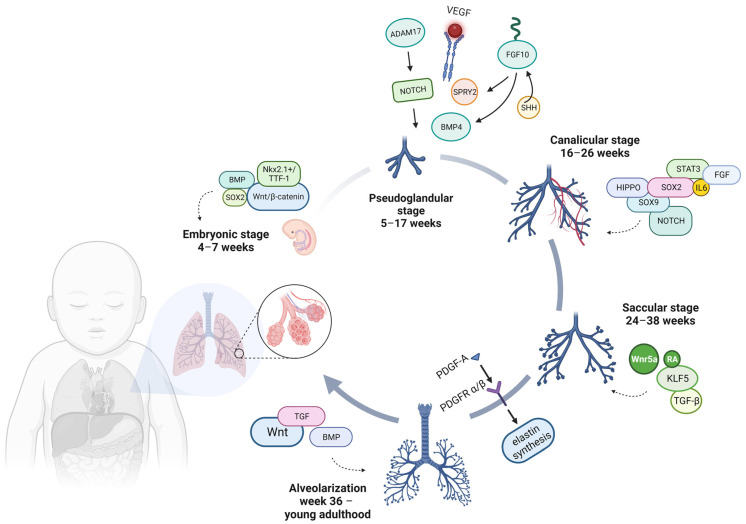
Simplified diagram depicting the sequence of signaling events that take place during human lung development stages. Created with BioRender.com (accessed on 8 November 2023).

A homeobox-containing transcription factor, NKX2.1, also known as thyroid transcription factor-1 (TTF-1), is found in two of the numerous structures that form the endoderm of the gut and regulates the expression of pertinent functional genes within thyroid and lung tissues (Figure 1) [10,11]. In the lung, occurring on embryonic (E) day 9 in mice and around gestational day 28 in humans, the expression of NKX2.1 is present in all epithelial cells during the initial stages of pulmonary development but gradually becomes limited to type II pneumocytes and bronchiolar exocrine cells (club cells) [5].

Furthermore, research has demonstrated that Wnt signaling is pivotal in determining whether respiratory endoderm progenitors express NKX2.1+ during development [12]. Wnt signaling represents an example of a pathway recognized for its significance in early tissue morphogenesis [13]. When β-catenin is lost or when the Wnt inhibitor dikkopf1 is expressed in the lung epithelium following lung specification, it results in reduced development of the distal airway epithelium and an overall shift toward a more proximal lung development pattern [14,15].

The targeted deactivation of Bone Morphogenetic Protein (BMP) type I receptor genes, specifically Type-1A BMP receptor (BMPR1A) and Type-1B BMP receptor (BMPR1B), within the ventral endoderm results in the absence of the trachea and the presence of abnormal primary bronchi. Molecular investigations of these mutant specimens indicate a decrease in the ventral endoderm marker NKX2-1 and an extension of the dorsal markers’ sex-determining regions Y-box 2 (SOX2) and P63 into what would typically become the trachea and primary bronchi. The BMPR1A and BMPR1B signaling pathways serve dual roles in the initial stages of respiratory development: firstly, they facilitate the formation of the trachea by inhibiting Sox2 expression, and secondly, they confine the location where the lung bud begins to emerge [16].

#### 3.1.2. Pseudoglandular Stage: 5–17 Weeks

During this phase, the lung consists of numerous epithelial tubules enveloped by extensive areas of mesenchyme, resulting in a gland-like appearance. Cellular division becomes more active during this phase, particularly at the extremities of the tubes known as bronchial buds [17]. By the culmination of this stage, the development of the conducting airways, encompassing the terminal bronchioles, reaches its conclusion around 17 weeks into development. This results in establishing 12–17 generations of bronchial tubules in the upper lobes, 18–23 in the middle lobes, and 14–23 in the lower lobes [18]. The mechanisms responsible for branching are intricately controlled through extensive communication between the epithelial and mesenchymal components, all in a precise temporal and spatial manner. The expansion of lung buds, their branching, and the eventual cessation of bud growth are outcomes of the dynamic interplay of molecules like Sonic Hedgehog (SHH), fibroblast growth factor 10 (FGF10), Sprouty RTK Signaling Antagonist 2 (SPRY2), transforming growth factor beta (TGFβ), and BMP4 [19,20].

FGF signaling, with a particular emphasis on FGF10, plays a critical role in the process of branching morphogenesis. It was observed that although the trachea formed in Fgf10^−/−^ mice, they died at birth due to a lack of lung development and pulmonary branching morphogenesis being disrupted [21]. FGF10 is produced in the distal lung mesenchyme, specifically at the locations where branches form and extend. It exerts its influence in a paracrine manner on the surrounding epithelium [22]. This interaction triggers a signaling cascade that ultimately results in the expression of SPRY2, SHH, and BMP4. These molecules, in turn, govern the response and growth of the lung bud. In this stage, the vascular endothelial growth factor (VEGF) also has an essential role in the development and ongoing sustenance of the lungs.

Although a disintegrin and metalloproteinase 17 (ADAM17), which acts as a NOTCH activator, has a vital role in the regulation of lung inflammation by enhancing the permeability of epithelial and smooth muscle cells, promoting the secretion of inflammatory mediators, and facilitating the migration of leukocytes across endothelial barriers, a deficiency in ADAM17 results in decreased airway branching [23].

At the end of this phase, all pre-acinar airways up to the terminal bronchioles have completed their formation.

#### 3.1.3. Canalicular Stage: 16–26 Weeks

The canalicular phase is probably the earliest stage of lung development affected by premature birth. Representing a crucial juncture in the development of the respiratory system, during this period of lung development, the boundary between the conducting and respiratory units within the respiratory tree is delimited [24]. Consistent with BPD characteristics, this phase involves significant growth and refinement of the distal airspace structures and microvasculature [25]. During the canalicular stage, the acinar tubules and buds undergo elongation, division, and expansion, while the surrounding mesenchymal tissue gradually becomes thinner. This signifies the foundational and pivotal stage in forming the lung areas responsible for gas exchange.

In parallel, robust blood vessel development (angiogenesis) occurs in the mesodermal tissue surrounding the acinus, forming an intricate capillary network that initiates the establishment of the blood–air barrier. These cells undergo differentiation into type I pneumocytes, which constitute the alveolar wall. Simultaneously, in other overlying epithelial cells, the appearance of lamellar bodies associated with surfactant synthesis (composed of lipids and surfactant proteins A-C) occurs, distinguishing them as type II pneumocytes. Type I and type II pneumocytes, also known as alveolar epithelial cells type 1 (AEC1) and type 2 (AEC2), are primarily responsible for facilitating gas exchange, respectively, play a pivotal role in surfactant production, and also function as progenitor cells for AEC1 [26].

Although infants born at this stage can survive with intensive care techniques, survival rates are often compromised due to insufficient surface area for gas exchange and limited lung surfactant production by type II pneumocytes [27,28].

Remarkable progress has been made in the field of artificial placenta research. Benjamin Bryner and his team conducted pioneering research and have reported the outcomes in the survival of extremely premature lambs, born at gestational ages of 110–120 days, compared to the full term of 145 days for lambs. These lambs, roughly equivalent to 22–24 weeks of human gestation in terms of lung development, survived for a week with the assistance of an artificial placenta [29,30,31].

One of the primary pathways that play a crucial role in determining the fate of cells is the NOTCH signaling pathway. NOTCH transmembrane receptors facilitate communication between adjacent cells and play a central role in regulating the balance between the development of multiciliated and secretory cell lineages within the proximal airway epithelium. When NOTCH signaling is impaired or lost, it results in the absence of secretory cells and the predominance of multiciliated cells in the airways [32].

On the other hand, Hippo signaling halts branching morphogenesis and promotes alveolar differentiation by targeting β-catenin for degradation in the epithelial cells. This disruption of the Wnt-FGF feedback loop directs the differentiation of bud–tip epithelial progenitor cells [33]. Furthermore, both human and mouse miR-449a are upregulated in the distal lung, particularly during the canalicular stage. Their role is to facilitate the differentiation of the distal epithelium by regulating the expression of MYCN and the sex-determining region Y-box 9 (SOX9) [34].

Moreover, during this period, SOX2 plays a vital role in the branching morphogenesis of the bronchial tree and the differentiation of the airway epithelium [35,36]. Another group describes that in the alveolar niche, focusing on secretory and receptor activities, the growth and self-renewal of alveolar progenitor type 2 (AT2) cells are governed by a functional pathway involving interleukin-6 (IL-6), the signal transducer and activator of transcription 3 (STAT3), BMP, and FGF signaling [37].

#### 3.1.4. Saccular Stage: 24–38 Weeks

In the saccular stage, spanning roughly from the 24th week to the 36th week of gestation in humans, a crucial development occurs—the formation of the terminal saccule and the concurrent emergence of respiratory bronchioles. These bronchioles have a thin wall that is conducive to efficient gas exchange.

In the canalicular stage, lung capillaries have a significant proliferation, which aligns closely with the epithelium to establish the initial air–blood barrier [5]. Following this, during the saccular stage, the final generation of airways takes shape, culminating in numerous thin-walled primitive alveoli, also known as saccules. In this stage, the capillaries form a double layer within the cellular intersaccular septa [24].

The remarkable growth of the potential respiratory airspaces results in a reduction of the interstitial tissue, significantly influencing how capillaries are organized. With the continued enlargement of each saccule, blood vessels draw nearer and create a dual-layered capillary network that envelops each saccule. This progression plays a vital role in the formation of alveoli and the subsequent facilitation of gas exchange. Shortly thereafter, elastin begins to accumulate beneath the epithelium, which is a crucial step in priming the lungs for subsequent alveolar development.

As the surfactant system undergoes development and maturation during the saccular phase, the survival rate of extremely premature infants increases [38].

The distinct ability of SOX2 and SOX9 cells to undergo differentiation into bronchiolar and alveolar lineages, respectively, enables the differentiation between the conducting and respiratory airways by the end of the canalicular stage. This process contributes to pulmonary maturation during the saccular phase, setting the stage for the first breath at birth [39,40].

During the saccular phase of lung development, the differentiation of AEC1 cells is dependent on Wnt5a, a non-canonical Wnt ligand. A recent study employing primary epithelial cells isolated from human fetal lungs underscores the role of Wnt5a as a selective regulator that ensures the correct balance between AT1 and AEC2 cells throughout lung development [41]. A similar process occurring during development is highlighted by the absence of KLF5, which hinders lung maturation during the saccular stage of development [42].

Active TGF-β signaling, as determined by Smad2 phosphorylation, was observed in various locations, including the vascular and airway smooth muscle and the alveolar and airway epithelium, during the later stages of lung development. These findings indicate the essential role of active TGF-β signaling in facilitating normal late-stage lung development [43].

Retinoic acid (RA) signaling stands as one of the paramount biological pathways in the natural world, initiated when RA interacts with nuclear receptors that regulate gene expression [44]. During the late saccular period, on the one hand, a reduction in the expression regarding the levels of the RA receptor-alpha (RARα) transcript is detected, and on the other hand, there is a notable increase in the expression of RA receptor-beta (RARβ), which closely aligns with the induction of AEC1 and AEC2 [45]. This suggests a potential role for RARβ in the preparation of the lungs for the alveolarization process.

#### 3.1.5. Week 36—Young Adulthood: Alveolarization

Alveolar formation is thought to occur from 36 weeks of gestation and persists until three years after birth, with most divisions occurring in the first six months of life. Moreover, as maturation progresses, the two-layered capillary network fuses into a single network, each network being intimately associated with two adjacent alveoli [24].

Starting in the third year of life, the enlargement of the lungs results primarily from the increase in the number of alveoli. According to some studies, this complex alveolarization process ends by the age of 8 years, and further lung growth occurs mainly by enlarging the individual alveoli. However, advances in imaging technologies and meticulous stereological analysis suggest that the process of alveolar formation continues into young adulthood, around age 21 [5,46]. As we mentioned, alveologenesis is a complex process characterized by significant cellular and tissue restructuring, ultimately forming a spacious surface for gas exchange. These remodeling activities encompass the ultimate refinement and maturation of AEC2 cells, the production of surfactant, the flattening of AEC1 cells, and the differentiation of mesenchymal components. The platelet-derived growth factor A chain (PDGF-A) and its receptors (PDGFRα/β) have been demonstrated to have significant involvement in the differentiation of myofibroblasts and the synthesis of elastin [47,48].

Bundles of elastin, deposited by myofibroblasts, act as a structural framework to constrain the expanding alveolar cells as they transform into sacculi during lung development. This remodeling process is primarily orchestrated by mesenchymal cells, particularly alveolar myofibroblasts (MYF), which are activated by PDGF-A and SHH signaling. Additionally, the contractile properties of these MYFs contribute to the physical shaping of the alveolus. Furthermore, Wnt-responsive AEC2 cells receive Wnt ligands (signaling reactivated during alveologenesis) from the adjacent mesenchymal cells, increasing proliferation during this phase. This heightened proliferation is essential for boosting surfactant production, a critical step in the transition to air breathing [49].

In addition, the TGF superfamily of transforming growth factors and BMP, the alternative branch of the TGF-β superfamily, have also been described as being involved in alveolarization and, respectively, postnatal lung maturation (specifically, in the production of surfactant in newborns as they adapt to breathing in the external environment) [50].

### 3.2. Expression of microRNAs during Lung Development

MicroRNAs (miRNAs) typically consist of around 21 to 24 nucleotides and hold significant importance as they play a crucial role in the post-transcriptional regulation of gene expression [51]. In numerous scenarios, the signaling molecules discussed earlier are additionally controlled by specific types of RNAs to ensure precise coordination of development in both temporal and spatial dimensions [52]. The indication that miRNAs are involved in lung development is supported by experiments involving mice, where Dicer, a key miRNA-processing enzyme, was conditionally knocked out in lung epithelial cells. These mice displayed a notable deficiency in epithelial branching, highlighting the crucial regulatory function of miRNAs in shaping the morphogenesis of lung epithelial tissue [53]. An abnormal expression of Fgf10 may also contribute to the morphological aberrations in Dicer. During the early stages of mouse embryonic development, miR-127 exhibited an upregulation at E6.5 and E7.5 [54]. Additionally, miR-127 exhibits significant expression levels within the endoderm and primitive streak. When miR-127 is overexpressed, it results in an elevated expression of mesendoderm markers, whereas inhibiting miR-127 leads to a reduction in their expression. Moreover, the influence of miR-127 on mesendoderm differentiation is diminished when Lefty2 is overexpressed.

Early in lung development, miRNAs like miR-302/367 and miR-17∼92 promote lung epithelium proliferation while suppressing differentiation. In contrast, during later stages, miRNAs such as miR-34/449 and miR-375 decrease proliferation and actively support differentiation in the lung [5]. miR-326 and Arrb1 exhibit an overexpression pattern in mesenchymal tissue during lung development, akin to that of Ptch1. Likewise, miR-326 plays a regulatory role in the Shh pathway by targeting Smo and Gli2 [55].

The miR-17 family of miRNAs consists of three sets of paralog clusters, namely, miR-17-92, miR-106a-363, and miR-106b-25. miR-17-92 cluster overexpression has been identified as a pivotal factor in regulating both the differentiation and proliferation of lung progenitor cells [52]. Deletions of the miR-17-92 cluster have been demonstrated to result in the development of smaller embryos that exhibit postnatal lethality, marked by significantly hypoplastic lungs. Additionally, when both the miR-17-92 cluster and its paralog miR-106b-25 were simultaneously deleted, the mice displayed an even earlier onset of lethality before reaching embryonic day 15 [56].

In the course of transitioning from the pseudoglandular to canalicular phase, when the distal respiratory segment of the airways initiates its formation, a microarray analysis revealed that the most notably differentially expressed miRNA was miR-449a, with upregulation during the 18–20-week period. Similarly, results were found in the mouse embryonic lung [57]. When miR-449a was inhibited in mouse lung cultures (at E16.5—the end of the pseudoglandular stage), overexpressed mRNA levels for MYCN and SOX9 were observed, as well as an increase in protein levels for Ki-67 and SOX9, particularly in the distal epithelial region [34]. Regarding the regulation of pulmonary surfactant secretion, miR-150, miR-375, and miR-26a have been documented to play a significant role [58,59]. Experimental research has demonstrated their significant role in lung development as well as the synthesis and metabolism of pulmonary surfactant [60].

## 4. Pulmonary Surfactant Metabolism Dysfunction

The transition from the fetal to neonatal period involves a critical physiological process, which includes the production of pulmonary surfactant. Surfactant, a complex mixture of phospholipids (comprising a significant proportion; these components make up approximately 80% to 85% of the overall mass), neutral lipids (5–10%), and specific proteins (approximately 10%), has the role of preventing the collapse of the alveoli in the lungs by reducing the surface tension.

Insufficient pulmonary surfactant production due to immaturity is a primary factor contributing to RDS. The classic clinical depiction of RDS has undergone alterations over time in response to evolving treatments. Nowadays, instances of radiographic “ground glass with air bronchograms” are infrequent due to the widespread use of early surfactant therapy and continuous positive airway pressure in the initial stages of treatment. The reliance on blood gas analysis for defining and diagnosing the condition has diminished as clinicians have shifted towards a more practical approach, which involves assessing the need for surfactant therapy based on a clinical evaluation of factors such as the respiratory effort of the patient and early oxygen requirements [61].

Apart from prematurity, various other factors may contribute to RDS development: non-genetic and genetic factors. Maternal or perinatal factors and characteristics of the newborn, such as advanced maternal age, oligohydramnios, chorioamnionitis, delivery method, gender, birth sequence, and external environmental factors, are described as non-genetic risk factors [62,63,64,65,66]. Based on the penetrance of the gene’s variants, the etiology may be determined only by the genetic factors (if the penetrance is high) or by the association of genetic (low penetrance) and non-genetic factors [67].

Some gene variants have been identified in a subset of challenging cases of RDS, including those affecting genes responsible for surfactant protein B (SP-B), surfactant protein C (SP-C), and the adenosine triphosphate-binding cassette transporter A3 (ABCA3). Certain variations linked to surfactant composition, pulmonary maturation, and inflammatory response may be potential candidates for the onset of RDS. Pathogenic variants in genes responsible for *SFTPB* and *ABCA3* lead to severe and frequently fatal lung diseases, usually inherited in autosomal recessive patterns [68,69]. Furthermore, pathogenic variants of *SFTPC* and some of the *ABCA3* genes are recessive and dominantly inherited and have been linked to chronic interstitial lung disease (ILD) in term newborns, children, and adults [70].

### 4.1. ABCA3 Gene Variants

The lipid transporter known as ATP binding cassette, class A3 (ABCA3), is a strongly preserved multi-membrane-spanning protein, serving as a pivotal regulator in maintaining the balance of pulmonary surfactant. The significance of ABCA3 in maintaining surfactant balance is emphasized by strong evidence indicating that full-term neonates carrying biallelic variants in the *ABCA3* gene experience surfactant deficiency, leading to life-threatening respiratory distress [71,72]. Within the spectrum of genetic factors contributing to RDS, the most prevalent is the *ABCA3* E292V variant, a Type II missense substitution. This variant affects the process of pulmonary surfactant assembly in pneumocyte II lamellar bodies [73]. It is frequently detected in a compound heterozygous state in conjunction with other Type I or Type II variants [74,75]. In mechanistic studies conducted in vitro, researchers have categorized *ABCA3* variants into three classes. Type I variants, exemplified by L101P, L982P, Q1591P, and L1553P, lead to protein misfolding and disrupt the normal intracellular trafficking of the transporter. Type II variants are located in close proximity to the catalytic domains of the transporter, and examples include E292V, N568D, E690K, and T1114M. Although these variants undergo normal trafficking to lamellar bodies or lysosomal-like organelles, they lead to a deficiency in ATP hydrolysis, resulting in impaired lipid transfer [76]. Type III variants frequently result in a more pronounced and severe clinical phenotype [77].

The available evidence suggests that individuals with variant homozygous or compound heterozygous genotypes often experience critical illness [78,79,80] since the level of the ABCA3 protein decreases, or its function is affected following a recessive pattern. Considering this pattern, special attention should be paid to patients with compound heterozygous genotypes [81,82,83]. It is also worth noting the importance of choosing the correct method or technique for testing, in particular, this type of genotype, with sequencing being mandatory. Depending on the genetic alteration, some patients may exhibit a more diverse range of clinical presentations and outcomes. However, the long-term overall survival rate (>5 years) for individuals with *ABCA3* variants was found to be less than 20% [84].

Table 1 displays the classification of pathogenic variants with high penetrance in relation to the clinical progression of patients. Besides those variants, there are single nucleotide polymorphisms. For example, R288K has been linked to pediatric respiratory conditions in prior studies [81]. Additionally, it was found to be three to four times more prevalent among infants of European descent who experienced neonatal respiratory distress, despite predictions by the Polyphen and SIFT algorithms indicating that it is benign and well tolerated [85]. In contrast, another study proposes that having heterozygous R288K variants in *ABCA3* alone may not be sufficient to trigger interstitial lung disease in the absence of additional contributing factors [86]. Many of the variants are distinctive, encompassing more than 300 distinct variants documented in the scientific literature. This diversity poses challenges for genetic counseling within families [87].

Assessing the pathogenicity of newly discovered variants is of utmost importance, as it leads to the diagnosis, clinical management, and genetic counseling process. The categorization of different *ABCA3* gene variants based on their impact on the functionality of the ABCA3 transporter was made possible through a comprehensive approach (functional genomics, Western Blot, immunofluorescence, etc). This approach, which integrates molecular defect insights from in vitro and ex vivo research, allows the pinpointing of specific variant categories that may potentially benefit from small-molecule interventions aimed at restoring the ABCA3 transporter’s proper function [86].

### 4.2. SFTPB Gene Variants

Surfactant proteins B (SP-B) and C (SP-C) are highly hydrophobic proteins with low molecular weight that play a crucial role in enhancing the surface-tension-reducing capabilities of surfactant lipids. They are especially important for the ability of surfactant to adhere and disperse across the air–liquid interface. These proteins are essential components of commercially available replacement surfactants that are commonly employed in the treatment of premature infants suffering from RDS.

Changes in the expression of the *SFTPB* gene can cause pulmonary inflammation and severe respiratory failure, both through changes at the DNA level and through epigenetic changes. Pathogenic variants in the *SFTPB* gene result in SP-B deficiency and lead to a fatal neonatal respiratory disorder known as hereditary SP-B deficiency [101]. Newborns affected by hereditary SP-B deficiency, usually born at full term, experience severe respiratory failure shortly after birth, resembling the RDS seen in premature infants [96]. An analysis of 68 premature newborns born with a gestational age under 32 weeks and diagnosed with an unusually severe form of RDS revealed that a significant proportion (35%) of these premature newborns carried heterozygous rare or novel variants in surfactant protein genes *ABCA3*, *SFTPB*, and *SFTPC*.

### 4.3. SFTPC Gene Variants

A multitude of pathogenic *SFTPC* variants have been documented in various populations and among premature infants suffering from severe respiratory distress [102,103]. *SFTPC* variants, initially characterized by Nogee et al., were originally linked to an autosomal dominant inheritance pattern with varying levels of expression and penetration [104]. De novo variants are frequently observed as well [105,106,107].

Due to their high clinical expressivity, it is recommended to include surfactant variants in the list of potential diagnoses, even when dealing with respiratory infections that are much more commonly associated with certain seasons or pandemics. This idea is sustained by a case report where genetic testing of a 7-day-old male newborn from an uneventful pregnancy and delivery with no family history of illness revealed a heterozygous de novo variant in the *SFTPC* gene [108].

### 4.4. NKX2-1 Gene Variants

The NK2 homeobox-1 (*NKX2.1*) gene encodes the thyroid transcription factor-1 (TTF-1) and serves as a pivotal contributor to the development and function of the lung, thyroid, and central nervous system. Pathogenic variants in this gene give rise to a rare form of progressive respiratory failure, which is linked to changes in surfactant production, structure, and balance, influencing the expression of associated genes, including *SFTPB*, *SFTPC*, and *ABCA3*. The underlying molecular mechanisms for these alterations are diverse and relatively uncharted [109]. Currently, there are no epidemiological estimates available for NKX2-1 haploinsufficiency [110]. Nearly all the pathogenic variants of *NKX2-1* so far have been newly described: as spontaneous variants (de novo) or with autosomal dominant inheritance patterns [111,112].

The patients with pathogenic variants exhibited a range of clinical manifestations, including benign hereditary chorea, hypothyroidism, chronic ILD, and respiratory distress syndrome, which is also referred to as brain–lung–thyroid syndrome [113,114].

In addition to gaining a deeper understanding of the progression of infants and children with varying genotype–phenotype associations, there is a need for research on the factors, both genetic and environmental, that extend survival without the necessity of transplantation. Furthermore, functional investigations into the mechanisms underlying the disease and randomized clinical trials of pharmacotherapeutics are required to discern whether the anecdotal responses to empirical therapies are attributed to the improvement of the condition or align with the natural course of the disease [115].

More variants associated with the risk of RDS were also found in the surfactant protein A1 (*SFTPA1*) gene, the surfactant protein A2 (*SFTPA2*) gene, the surfactant protein D (*SFTPD*) gene, the nitric oxide synthase 3 (*NOS3*) gene, and the lysophosphatidylcholine acyltransferase 1 (*LPCAT1*) gene [116,117,118,119,120].

## 5. miRNAs as Biomarkers for RDS

Genomic research provides potent tools to explore which plasma or imaging markers might be causative in the development or fatality associated with RDS. This information could then be used to enhance the efficiency of preclinical trials, select high-risk patients for clinical trials, or even identify specific RDS subtypes suitable for testing targeted therapies. Achieving precision medicine in RDS prevention and treatment appears to be a realistic objective for the next 50 years of RDS research. Changes in DNA rarely cause RDS, but epigenetics is frequently implicated in disease etiology and evolution. Any of the miRNAs discussed in the human lung development chapter can be involved in RDS, but in this section, we will focus on secondary pathways, like the inflammatory response.

**Figure 2:**
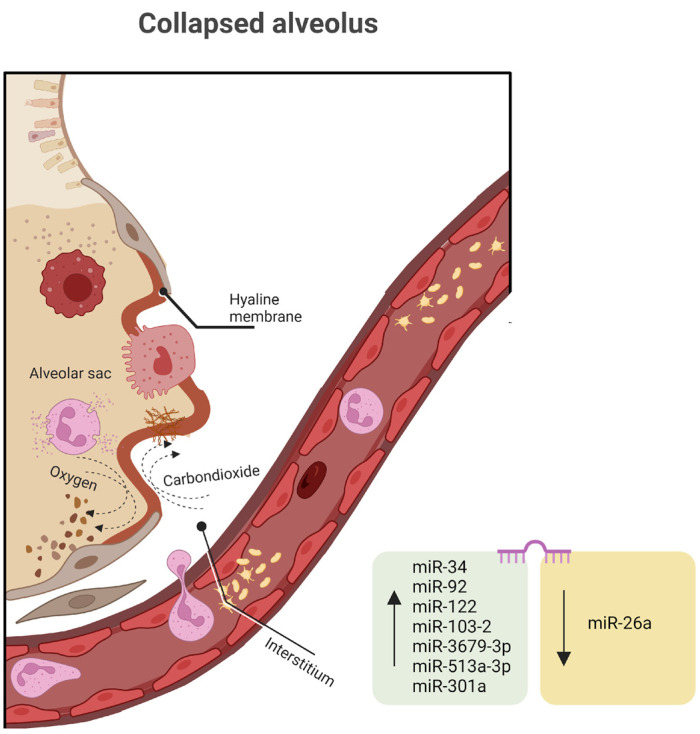
miRNA dysregulation in neonatal respiratory diseases syndrome (↑ represents upregulation, and ↓ represents downregulation). Created with BioRender.com (accessed on 8 November 2023).

Several miRNAs discovered in both high-throughput screening studies involving RDS patients and preclinical animal models have indicated the potential involvement of miRNAs in the development of RDS (Figure 2). These miRNAs appear to regulate inflammatory pathways and modulate immune responses in the context of RDS pathophysiology. For example, recent research has demonstrated that miR-34a has the capacity to modulate inflammatory responses [121]. According to the available literature, various disease mechanisms can lead to an augmentation of the alveolar epithelial barrier and an increase in pulmonary vascular endothelial permeability in RDS patients, which subsequently leads to the development of pulmonary edema [122]. Angiopoietin (Ang) plays a significant role in regulating the proliferation and apoptosis of vascular endothelial cells [123]. Ang represents a group of growth factors primarily secreted by endothelial cells. This group includes various subtypes, such as Ang-1, Ang-2, Ang-3, Ang-4, and others. Among these, Ang-1 and Ang-2 have been the subjects of more extensive research in the context of inflammation. Studies have indicated that assessing the Ang-1 levels can be valuable in assessing the survival status of patients with acute respiratory distress syndrome [124]. Serum miR-34a, Ang-1, and the neonatal critical score exhibit strong associations with the severity and prognosis of neonatal RDS. These findings suggest that serum miR-34 may play a role in the development of neonatal RDS and could serve as a prognostic marker for children with RDS. Additionally, the serum level of Ang-1 and the NCIS score (analyze and observe illness indicators in infants) are closely tied to the severity of RDS in pediatric patients [125].

Similarly, recent research has revealed the expression of miR-92 and miR-122 in inflammatory lung conditions. They are actively engaged in numerous signaling pathways and diverse immune–inflammatory responses, therefore exerting a crucial regulatory influence on the onset and progression of respiratory distress syndrome [126,127].

An analysis conducted on a cohort of 20 infants diagnosed with RDS and 29 infants without RDS, all with a gestational age between 28 and 34 weeks, revealed 171 distinctively expressed miRNAs, consisting of two upregulated and seven downregulated miRNAs associated with RDS. Among these miRNAs, four were singled out for their notably higher fold changes when comparing the two groups. miR-103-2 is typically upregulated as a response to hypoxia and plays a role in regulating pyruvate and lipid metabolism. miR-3679-3p, miR-513a-3p, and miR-301a, which have received relatively limited research attention, also display significant expression levels as RDS pathology advances [128]. Previously, salivary miR-3679-3p expression was reported to be elevated in individuals with nasopharyngeal carcinoma, and miR-513a-3p has been identified as a sensitizer in human lung adenocarcinoma cells during chemotherapy by targeting glutathione S-transferase P1 (GSTP1), thereby promoting apoptosis [129,130].

The introduction of miR-26a mimics notably suppresses the protein expression of GSK-3β and triggers the Wnt/β-catenin signaling pathway, influencing inflammatory responses in neonatal rats with RDS. Additionally, miR-26a reduces the levels of pro-inflammatory markers, including tumor necrosis factor-alpha (TNF-α), interleukin-1β (IL-1β), and interleukin 6 (IL-6), along with RAGE (the receptor for advanced glycation end products), PAI-1 (plasminogen activator inhibitor-1), and HMGB1 (high mobility group box 1) [131]. In order to delve deeper into the in vivo biological functions of miR-26a, an attempt was made to create mice with double knockouts of miR-26a-1 and miR-26a-2 (referred to as miR-26a-1^−/−^/miR-26a-2^−/−^ mice) using the clustered regularly interspaced short palindromic repeat/CRISPR-associated protein 9 (CRISPR/Cas9) system [132]. While further investigations into the molecular mechanism are required, the lungs of the miR-26a-1/miR-26a-2 double-knockout mice displayed elevated quantities of mature cells, particularly AEC2, and an increase in pulmonary surfactant synthesis. These findings strongly suggest the proximal role of miR-26a in lung development and its impact on pulmonary surfactant production.

Initially, circulating miRNAs were applied as biomarkers in serum to investigate patients with diffuse large B-cell lymphoma [133]. Since then, numerous studies have documented miRNA dysregulation in various human diseases. To begin, techniques such as microarray profiling, real-time PCR arrays, and next-generation sequencing (NGS) were utilized for the initial assessment of circulating miRNAs, leading to the creation of distinctive miRNA signatures from bodily fluids. Furthermore, the recognition and validation of these aforementioned characteristics imply the possibility that those miRNAs may, to some extent, contribute to the development of RDS and potentially serve as valuable biomarkers for diagnosing RDS.

## 6. Therapeutic Targets

### 6.1. Lung Transplantation

Available treatments for monogenic pulmonary diseases are currently constrained in their specificity and scope. For numerous patients, a lung transplant may represent their sole chance of survival beyond the initial months of life. Nonetheless, individuals who undergo lung transplantation face a heightened risk of mortality and transplant-related complications when compared with recipients of other solid organ transplants [134]. The majority of infants who were critically ill at the time of transplantation and required mechanical ventilation (including high-frequency oscillatory ventilation) or ECMO (extracorporeal membrane oxygenation) received transplants due to surfactant protein B or ABCA3 deficiency [135]. Approximately 70% of patients may experience the development of renal insufficiency and hypertension as a consequence of the adverse effects of long-term immunosuppressive medications [136].

As an alternative to lung transplantation, a combination of tracheostomy and chronic ventilation, along with the use of empirical anti-inflammatory agents such as corticosteroids, azithromycin, hydroxychloroquine, and/or azathioprine, has been proposed for infants and children with *SFTPC* variants experiencing severe and prolonged respiratory failure. Some children have been reported to successfully wean off all respiratory support and have their tracheostomies removed over a period of 2 to 6 years [137,138]. The potential treatment effect of hydroxychloroquine on cases of *ABCA3* gene variants may be attributed to its anti-inflammatory properties or its ability to modify intracellular metabolism [139]. Corticosteroids have demonstrated the ability to increase the expression of ABCA3 in alveolar type II cells, while macrolides can effectively suppress the production of numerous pro-inflammatory cytokines and inflammatory mediators that play a role in the progression of interstitial fibrosis.

Despite the fact that infants with biallelic loss-of-function variants in the *SFTPB* and *ABCA3* genes phenotypically present with severe respiratory failure at birth and require lung transplantation, given their increased mortality up to 1 year of age, infants and children with other variants in the *ABCA3* gene, including missense, in-frame insertions or deletions, splice sites, or variants in the *SFTPC* and *NKX2-1* genes, show a more diverse association between genotype and phenotype and are less predictable [140,141].

### 6.2. Gene Therapy

In a preliminary gene addition investigation, human *SFTPB* was introduced into murine MLE12 cells using adenoviral vector delivery. These mouse cells, while maintaining characteristics akin to AT2 cells, exhibited initially low SP-B levels that experienced an increase following adenoviral gene transfer. When the identical adenoviral vector was administered to cotton rats, it induced a peak in human SP-B levels within 48–96 h post-administration, followed by a subsequent decline to undetectable levels [142]. Recombinant adeno-associated viral (rAAV)-vector-based gene therapy has been adapted for use in more than 100 clinical trials. AAV vectors have garnered attention due to their growing clinical use, especially since the FDA granted approval for Luxturna and Zolgensma, alongside encouraging research in the context of Duchenne muscular dystrophy. This is mainly because of its excellent safety profile, ability to target a wide range of tissues, stable transgene expression, and significant clinical benefit. Viral vectors find widespread use in respiratory-related applications.

Researchers have developed a strategically designed AAV6 capsid, which has shown efficacy in transducing lung epithelial cells, as demonstrated through imaging and flow cytometry analysis. When administered intratracheally, this vector delivers murine or human proSFTPB cDNA to SP-B-deficient mice, leading to the restoration of surfactant balance, the prevention of lung injury, and the enhancement of lung function. In contrast, untreated SP-B-deficient mice face fatal respiratory distress within two days. With gene therapy, the median survival period extends to over 200 days. Moreover, this vector is also capable of transducing human lung tissue, indicating its potential for clinical application in the treatment of this life-threatening condition [143].

Moreover, by employing an innovative technique for serotype screening, researchers have utilized a human 3D cell culture model of the lung, known as lung bud organoids (LBOs), termed alveolospheres, which are derived from human embryonic stem cells (hESC). In this study, they identified that naturally occurring rAAV2 and rAAV6 serotypes, as well as synthetic rAAV6 variants, exhibit an affinity for the human lung parenchyma. The presence of positive staining for surfactant proteins B and C in LBOs not only confirmed their identity as distal lung tissue but also indicated the potential suitability of these vectors for transducing alveolar type II cells [144].

Another study demonstrated successful gene editing of the *SFTPB* gene in a mouse model. They achieved this by utilizing a mouse model with SP-B expression controlled by a doxycycline-inducible promoter, delivering nuclease-encoding chemically modified mRNA, and introducing donor DNA through an AAV vector to insert a CAG promoter upstream of the *SFTPB* start codon. This intervention resulted in the mice’s extended survival. These findings offer valuable insights into the potential effectiveness of gene editing approaches for addressing SP-B deficiency [145].

Lentiviruses, commonly referred to as LVs, enjoy extensive use and have garnered FDA approval for clinical trials aimed at addressing various conditions, including SCID, chronic granulomatous disease, adrenoleukodystrophy, and beta-thalassemia [146,147]. In most cases, LV-based therapies for these diseases primarily entail the ex vivo modification of hematopoietic stem cells, which are then reintroduced into the body of the patient. Despite the notable achievements of ex vivo LV therapies, delivering these vectors to the lung remains a complex endeavor.

Currently, in gene therapy applications, three main types of engineered herpes simplex virus (HSV) vectors, namely amplicon HSV, replication-defective HSV, and replication-competent HSV, are utilized, capitalizing on the virus’ natural neurotropism. The key features of HSV vectors include their capacity to evade the immune system, their ability to deliver substantial DNA cargos and multiple genes, and their inherent or engineered cell-specific lytic properties [148].

In recent times, genome editing methods have emerged, such as zinc-finger nucleases (ZFNs), transcription activator-like effector nucleases (TALENs), and the CRISPR/Cas9 system. Nevertheless, the CRISPR/Cas9 system offers substantial advantages over ZFNs and TALENs [149]. These advantages include enhanced precision in targeting, the capability to disable multiple target genes simultaneously, more straightforward and less time-intensive experimental procedures, and the absence of restrictions related to species [150]. AAV is a frequent choice for transporting gene editing tools. This includes the utilization of a split-intein system to enable the simultaneous delivery of cassettes that exceed the AAV packaging capacity. Another approach involves delivering the smaller Staphylococcus aureus Cas9 along with a sgRNA in a single AAV vector, as demonstrated by Lau and Suh in 2017 [151]. When developing a gene editing treatment for any disease, it is essential to consider the potential need for an individualized approach, particularly in the case of the various pathogenic *SFTPC* variants responsible for interstitial lung disease.

### 6.3. miRNA-Based Treatment

Moreover, although miRNA-based treatments specifically designed for RDS are not currently available, miRNA-targeted therapies have progressed to phase II clinical trials for various medical conditions [152,153]. Given their involvement in the regulation of numerous biological processes, miRNAs are compelling candidates as potential targets for novel pharmacological interventions. Therapies aimed at miRNAs have been created or are currently in development for a range of inflammatory disease conditions. Therefore, future investigations may reveal distinctive miRNA profiles that could serve as biomarkers or as targets for pharmacological miRNA-based interventions. miRNA levels can be enhanced through the introduction of an miRNA mimic or an expression vector, typically using a plasmid or virus. Conversely, miRNA levels can be suppressed through the use of anti-miRNA oligonucleotides (AMOs), small molecular inhibitors targeting specific miRNAs (SMIRs), and miRNA sponges.

Although, to our knowledge, there are no data on miRNA-based therapies in neonatal respiratory distress syndrome, their role in other inflammatory processes has been documented. miR-126 is highly present in exosomes derived from human endothelial progenitor cells. The intratracheal delivery of these exosomes mitigated lung injury severity in a murine model of intratracheal lipopolysaccharide lung injury [154]. Likewise, enhancing miR-126 expression therapeutically has the potential to enhance pulmonary barrier function in cases of acute respiratory distress syndrome (ARDS). Another miRNA discovered to provide protective attributes is miR-146, with a role in suppressing the hyperactive inflammatory reaction [155]. miR-150 has been shown to offer protection by regulating apoptosis, inflammation, cytokine production, and metabolism by targeting AKT serine/threonine kinase 3 (AKT3), consequently inhibiting JNK signaling in A549 alveolar basal epithelial cells [156].

Furthermore, increasing evidence supports the utility of the lung ultrasound score (LUS) in neonatal intensive care, having a significant predictive capacity for important outcomes in neonatal RDS [157]. For preterm neonates with RDS, the trajectory of the LUS is influenced by gestational age, strongly linked with oxygenation status, and serves as a predictive indicator for bronchopulmonary dysplasia [158]. In some specific populations, the LUS proves to be a valuable, convenient, and noninvasive tool for monitoring respiratory health at the bedside [159]. This approach not only enhances the visualization of the lung apex and focal lung lesions but also allows for a more comprehensive assessment of lung injury severity. Consequently, it holds significance in predicting RDS prognosis and guiding clinical interventions. Currently, most research focuses on the clinical use of miRNA in adult ARDS, with a lack of relevant studies exploring the prognostic implications of miR-92 and miR-122 in pediatric ARDS. In a study on pediatric patients, the increased expression of serum miR-92 and miR-122 was correlated with the severity and prognosis of ARDS children, and in association with the LUS, it can predict the prognosis of ARDS children [160]. Furthermore, the LUS and the plasma expression level of miR-21-3p exhibit a correlation with the severity and prognosis of patients with acute lung injury. When used in combination, these two factors hold significant value in assessing the prognosis of patients with acute lung injury [161]. These investigations are in the initial exploratory phase, and additional prospective studies are required to conclusively establish the significance of miRNAs in conjunction with the LUS for neonatal patients with RDS.

Before stating our conclusions, we had to mention that the strengths and novelty of our article consist in the fact that our study brings together the descriptive and functional genomics of RDS. However, there are several limitations, one of them being the fact that currently, few functional or case–control studies are published; in order to decipher better (in terms of clinical consequences) our genome, more studies are needed in this field.

## 7. Conclusions

Collectively, it becomes evident that signaling pathways play a significant part in both lung development and the advancement of various lung diseases. Gaining a more profound comprehension of the factors that govern lung morphogenesis is essential for effectively addressing congenital and neonatal respiratory disorders. The rapid progress in molecular and cellular biology techniques is significantly aiding the investigation of signaling pathways within lung biology. Enhancing our understanding of the precise signaling mechanisms within the lungs holds the promise of enabling the creation of more targeted therapies, potentially offering therapeutic benefits to individuals affected by lung diseases.

Given their pivotal roles in surfactant function and metabolism, common variations in the *ABCA3*, *SFTPB*, *SFTPC*, and *NKX2.1* genes have been detected in certain challenging cases of RDS. Pathogenic variants in genes associated with *SFTPB* and *ABCA3* result in severe and often fatal lung diseases, typically inherited in an autosomal recessive manner. Contrarily, pathogenic variants in *SFTPC* and certain *ABCA3* genes can be inherited in both recessive and dominant patterns and have been linked to ILD in full-term newborns, children, and adults. When assessing patients presenting with symptoms of surfactant deficiency or dysfunction, it is crucial to take into account various factors, including non-genetic or environmental influences.

To some extent, miRNAs may play a role in the development of RDS and could serve as valuable biomarkers for diagnosing RDS. Moreover, gene-based therapies for surfactant dysfunction disorders arising from pathogenic variations in *SFTPB*, *SFTPC*, and *ABCA3* treatment are a realistic objective of RDS research. The combination of genomic approaches, including molecular pathway analyses, along with robust genetic association studies involving large and varied populations, has the potential to provide new and valuable insights in the times to come. Understanding the role of rare genetic variants, such as those in surfactant protein genes, in severe neonatal lung diseases in very preterm infants is imperative for guiding clinical practice. Elucidating the interplay between phenotype and genotype in these diseases could enhance diagnostic capabilities.

## Figures and Tables

**Table 1:** Variants in surfactant synthesis genes described in patients with clinical surfactant deficiency.

Gene	ID	HGVS Coding	HGVS Protein	Coding Impact	Functional Effect	Classification *	Treatment	Clinical	Reference(s)
*ABCA3*	rs149989682	c.875A>T	p.Glu292Val	missense	affects lamellar body formation; decreases vesicle formation	Pathogenic/Likely pathogenic	N/A	IPF/DPLD	[74]
	rs121909182	c.302T>C	p.Leu101Pro	missense	loss of proteolytic cleavage; loss of ATP hydrolysis activity; decrease in ATP binding in vitro	Pathogenic	N/A	Death, neonatal period	[68]
	rs1402761450	c.2945T>C	p.Leu982Pro	missense	loss of intracellular vesicle membrane location; loss of proteolytic cleavage;	Not reported	N/A	Death, neonatal period	[68]
	rs28936691	c.4772A>C	p.Gln1591Pro	missense	loss of intracellular vesicle membrane location; loss of proteolytic cleavage;	Pathogenic	N/A	ILD	[68]
	rs121909183	c.4658T>C	p.Leu1553Pro	missense	loss of intracellular vesicle membrane location; loss of proteolytic cleavage;	Pathogenic	N/A	Death, neonatal period	[68]
	rs121909184	c.1702A>G	p.Asn568Asp	missense	loss of ATP hydrolysis activity; decreases ATP binding in vitro; affects multivesicular bodies’ and lamellar bodies’ development; loss of phosphatidylcholine transport;	Pathogenic	N/A	Died after lung transplantation	[68]
	rs891579143	c.3341C>T	p.Thr1114Met	missense	N/A	Not reported	N/A	N/A	[70]
	N/A	c.2068G>A	p.Glu690Lys	missense	N/A	Pathogenic	N/A	Severe RDS Died	[88]
	N/A	c.1444C>T/ c.1111+1G>A ^C^	p.Gln482Ter/ Non coding	nonsense	N/A/ splicing, disruption in surfactant metabolism	Pathogenic/N/A	N/A	Died, 1 year and 1 month	[86,89]
	N/A	c.3862+1G>C	Non coding	N/A	splicing	N/A	iNO, PS, steroids	Died, 45 d	[83]
	N/A	c.1310T>C	p.Leu437Pro	missense	N/A	Likely pathogenic	MV, PS, inotropes, PDE inhibitors	RDS/PPHT Died, 100 d	[90]
	N/A	c.1142 T>G/ c.731G>T ^C^	p.Leu381Arg/ pArg244Met	missense	N/A	N/A	PS, azithromycin	Death—neonatal period	[91]
	N/A	c.3445G>A	p.Asp1149Asn	missense	N/A	Uncertain significance	MV, PS, triple therapy	Lethal RDS	[92]
	rs774112981	c.746C>T	c.746C>T	missense	N/A	Pathogenic	MV, PS, triple therapy	Lethal RDS Died, 53 d	[93]
	rs1298655924/N/A	c.2883C>T/ c.4885_4886insG ^C^	p.Gly961Gly/ p.Ala1629GlyfsX15	synonymous/ frameshift insertion	N/A	Likely pathogenic/N/A	CPAP/HFNC, steroids	Lung transplant	[94]
	rs751061681	c.604G>A/c.4036-3C>G ^C^	p.Gly202Arg	missense	N/A/splicing	N/A	N/A	Severe RDS Died, 99 d	[87]
*SFTPB*	rs779795223	c.361_362insAA	p.Pro121GlnfsTer95	frameshift	N/A	Pathogenic	MV, PS, atb, steroids	Died, 5 months	[95]
	N/A	c.541_552del12	Pro181_Leu184del	deletion	splicing	N/A	CPAP, PS, MV, Steroids	Died at 6 months	[96]
	rs138729391	c.394 T>C	p.Phe120Val	missense	N/A	N/A	MV, PS	Alive at 3 months	[96]
*SFTPC*	rs121917834	c.218 T>C	p.Ile73Thr	missense	N/A	Pathogenic	N/A	N/A	[97]
	rs76821562	c.547T>C	p.Cys183Arg	missense	N/A	N/A	MV, PS, atb, diuretics, IgVena	RDS	[98]
	N/A	c.1285+652_c.1468–3317del	N/A	deletion	N/A	Pathogenic	MV	Severe RDS	[99]
*NKX2.1*	rs758137643	c.334G>T	p.Gly112Ter	missense	N/A	Pathogenic/Likely pathogenic	oxygen therapy	N/A	[100]

* Clinvar database, https://www.ncbi.nlm.nih.gov/clinvar (accessed on 6 November 2023), and literature searching. ^C^, compound heterozygous; IPF, idiopathic pulmonary fibrosis; DPLD, diffuse parenchymal lung disease; RDS, respiratory distress syndrome; ILD, interstitial lung disease; PS, pulmonary surfactant; MV, mechanical ventilation; CPAP, continuous positive airway pressure; HFNC, high-flow nasal cannula; Atb, antibiotics; iNO, inhaled nitric oxide; steroids, systemic steroids; triple therapy, Methylprednisolone, Azithromycin, Hydroxychloroquine; PDE inhibitors, phosphodiesterase inhibitor; IgVena, human normal immunoglobulin; N/A, not available.

## Data Availability

Not applicable.
